# Sleep deprivation alters choice strategy without altering uncertainty or loss aversion preferences

**DOI:** 10.3389/fnins.2015.00352

**Published:** 2015-10-06

**Authors:** O'Dhaniel A. Mullette-Gillman, Yoanna A. Kurnianingsih, Jean C. J. Liu

**Affiliations:** ^1^Department of Psychology, National University of SingaporeSingapore, Singapore; ^2^SINAPSE Institute, National University of SingaporeSingapore, Singapore; ^3^Neuroscience and Behavioral Disorders Program, Centre for Cognitive Neuroscience, Duke-NUS Graduate Medical SchoolSingapore, Singapore; ^4^Division of Social Sciences, Yale-NUS CollegeSingapore, Singapore

**Keywords:** sleep deprivation, decision making, preferences, strategy, risk, ambiguity, loss aversion

## Abstract

Sleep deprivation alters decision making; however, it is unclear what specific cognitive processes are modified to drive altered choices. In this manuscript, we examined how one night of total sleep deprivation (TSD) alters economic decision making. We specifically examined changes in uncertainty preferences dissociably from changes in the strategy with which participants engage with presented choice information. With high test-retest reliability, we show that TSD does not alter uncertainty preferences or loss aversion. Rather, TSD alters the information the participants rely upon to make their choices. Utilizing a choice strategy metric which contrasts the influence of maximizing and satisficing information on choice behavior, we find that TSD alters the relative reliance on maximizing information and satisficing information, in the gains domain. This alteration is the result of participants both decreasing their reliance on cognitively-complex maximizing information and a concomitant increase in the use of readily-available satisficing information. TSD did not result in a decrease in overall information use in either domain. These results show that sleep deprivation alters decision making by altering the informational strategies that participants employ, without altering their preferences.

## Introduction

Total sleep deprivation (TSD) has been found to induce cognitive impairments and reduce the ability to make good decisions and judgments. The effects of TSD on behavior range from alterations of emotional processing (Yoo et al., 2007; Killgore et al., 2008; Gujar et al., 2011; see Killgore, 2010 for review), the desirability of food options (Greer et al., 2013), decision making across multiple domains (Harrison and Horne, 2000), and can even increase the likelihood of unethical behavior (Barnes et al., 2011). Recent studies indicate that a large percentage of people regularly suffer from sleep loss globally (Centers for Disease Control and Prevention (CDC), 2011). As such, it is important to understand how TSD influences economic decision making.

TSD has been shown to alter economic decision making across various tasks. For example, sleep-deprived persons have been reported to show an increase in effort discounting (Libedinsky et al., 2013), a shift in behavior from preventing losses to pursuing gains (Venkatraman et al., 2011), a change in the willingness to take risks on the Balloon Analog Risk Task (BART) (Acheson et al., 2007; Killgore, 2007), and poorer performance on the Iowa Gambling Task (IGT) (Killgore et al., 2006). However, it is unclear whether these alterations result from specific alterations of economic preferences, or from alterations of other cognitive aspects of the decision-making process. Accordingly, in this study we sought to specify the effects of sleep deprivation on economic decision making, focusing on preferences (uncertainty and loss aversion) and the information participants utilize to make their decisions (strategy), through a task that controls for potentially-confounding effects on other cognitive domains (such as learning).

Our first goal was to investigate whether sleep deprivation alters uncertainty and loss aversion preferences. Uncertainty preferences quantify how participants alter the valuation of a gamble due to an unknown probabilistic outcome. This can relate to risk (gambles of known probabilities), or ambiguity (gambles with unknown probabilities) (Knight, 1921; Ellsberg, 1961; Camerer and Weber, 1992). Empirical evidence has shown that people tend to be risk averse when making decisions about gains and risk seeking when making decisions about losses (Prospect Theory, Kahneman and Tversky, 1979; Kahneman, 2003). Given such clear behavioral differences between gains and losses, it is important to dissociably investigate the gains and losses domains.

The effects of TSD on uncertainty preferences have only been examined in a small number of experimental studies. Most such studies have focused on risk preferences in the gains domain and have found no modulation by TSD (Acheson et al., 2007; Venkatraman et al., 2007; Menz et al., 2012). Utilizing a task that involved altering 5-outcome gambles (involving both possible gains and losses within each trial) Venkatraman et al. (2011) suggested increased risk-seeking preference under TSD. One study by McKenna et al. (2007) explored choices to risky and ambiguity options in both the gains and losses domains, finding decreased risk aversion in the gains domain and decreased risk seeking in the losses domain. In this study, we sought to directly test the effects of TSD on risk preferences (independently in the gains and losses domains), while separately assessing alterations of loss aversion or strategy, and avoiding potential confounds (such as learning effects).

Loss aversion refers to the relative weighting of potential gains and losses in decision making, with the average person weighing potential losses approximately twice as strongly as potential gains (Tversky and Kahneman, 1992; Tom et al., 2007). Venkatraman et al. (2011) found neural evidence that TSD may alter economic choice behavior from “defending against losses to seeking increased gains,” which would suggest decreased loss aversion. However, they also specifically tested and found no alteration in loss aversion within their task. Here, using a task specifically designed to measure loss aversion (Tom et al., 2007), we directly test the hypothesis that sleep deprivation produces a decrease in loss aversion, either by decreasing the weighting of losses, increasing the weighting of gains, or both.

The final goal of this study was to investigate whether TSD alters the strategy with which participants engage with the available choice information. Our choice strategy metric quantifies the differential utilization of available information in decision making by contrasting between a simple satisficing strategy (the probability of winning the gamble) and a more cognitively-effortful maximizing strategy (determining the relative expected value of each of the available options) (Kurnianingsih et al., 2015; Mullette-Gillman et al., 2015). To date, no study has investigated the effects of TSD on such strategy utilization during economic decision-making. We hypothesized that TSD would result in a decrease in the use of maximizing information (e.g., calculated expected value information) with an increase in the use of readily-available satisficing information (e.g., probability information).

Recent studies have indicated that TSD may result in a decrease in the ability to process available information, including reduced visual short term memory information processing (Chee and Chuah, 2007), limited selective attention (Lim et al., 2010), reduced processing of peripheral information (Kong et al., 2012), and reduced rapid picture processing (Kong et al., 2014). This presents a secondary hypothesis for us to examine with our strategy analyses—whether TSD produces an overall decrease in the use of available information.

In this study, we examined how TSD alters economic preferences and choice strategy using three incentive-compatible decision-making tasks: (1) the gains choice task, (2) the losses choice task, and (3) the loss aversion task. We hypothesized that TSD would not alter uncertainty or loss aversion preferences, but would result in alterations of the information participants relied upon to make their decisions.

## Materials and methods

### Subjects

Data were collected from 29 members of the National University of Singapore community (17 males; age range = 19–26 years, mean ± SD = 21.66 ± 1.88 years). Participants selected for this study indicated that they: (1) had good habitual sleep (sleep duration of 6.5–9 h daily, sleeping before 00:30 and waking before 09:00), (2) were not of an extreme chronotype (as assessed by an abbreviated version of the Horne-Öbsterg Morningness-Eveningness questionnaire; Horne and Ostberg, 1976), (3) had no history of sleep, neurological, or psychiatric disorders, (4) were non-smokers, and (5) drank fewer than three caffeinated drinks per day. Additionally, participants' sleep patterns were monitored throughout the duration of the study through the use of wrist actigraphy (Actiwatch, Philips Respironics, USA); only those who evidenced good habitual sleep were included. All participants provided written informed consent in compliance with a protocol approved by the National University of Singapore Institutional Review Board, and in accordance with the Declaration of Helsinki.

### Study procedure

Participants made three visits to the lab, each scheduled 1 week apart. In the first visit, participants were briefed about the study protocol, trained on study tasks, and given a wrist actigraph to be worn throughout the study.

Participants then completed the rested wakefulness (RW) and total sleep deprivation (TSD) sessions, with session order counterbalanced across participants. All participants indicated that they had not consumed any medication, caffeine, nicotine, or alcohol for at least 24 h prior to each session.

For the TSD session, participants arrived at the lab at 19:00 the night before the experiment. Throughout the night, participants were monitored to ensure they kept awake and engaged only in sedentary activities. Participants also completed hourly assessments of vigilance (the 10-min Psychomotor Vigilance Task, PVT; Dinges et al., 1997).

For the RW session, participants arrived at the lab at 20:30 and were given 9 h of sleep opportunity. Participants performed one assessment of vigilance and of subjective sleepiness upon waking up.

**Figure 1:**
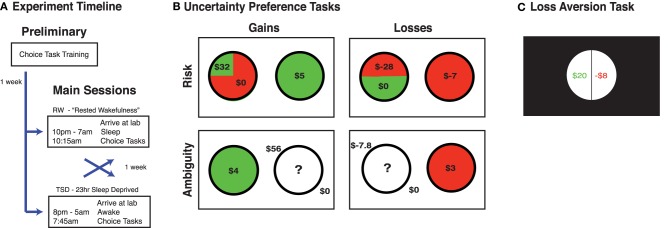
**Experimental design. (A)** Participants attended 3 sessions, 1 preliminary session followed by 2 main sessions, which were each ~1 week apart. The order of the RW and TSD session was counterbalanced across participants. **(B)** Example trials for the gains choice task and the losses choice task. **(C)** Example trial for loss aversion task. For all the monetary decision making tasks, resolution of one trial was given at the end of the experiment to determine participants' payment. No outcome resolutions were given during any tasks until completion of the final experimental session.

In the morning, our economic tasks began at 10:15 for the RW session, and 7:45 for the TSD session (see Figure 1A); as such, the effects described here represent the interaction of circadian and homeostatic factors. As part of a larger study, participants also completed additional computerized tests, behavioral questionnaires, and functional magnetic resonance imaging sessions (not reported here).

All participants completed three tasks in the following order: gains choice task, losses choice task, loss aversion task. At the beginning of the preliminary session, participants were informed that their final monetary payment would be adjusted by $0–$30 based upon the choices that they make. At the end of the entire experiment, we would randomly select one trial from each of the three tasks from each main session (6 trials total), resolve their choices, and pay them an unspecified percentage of the total funds they accumulated (which was 33%). Importantly, participants were reminded to treat each trial as the one that mattered, as it could be the one randomly selected and resolved. For the loss aversion task, participants were told they were receiving a $20 endowment (see task description), which was added to their accumulated funds, and helped ensure that participants ended up with a positive final accumulation. No trials were selected, nor gambles resolved, until the conclusion of the entire experiment to eliminate possible alterations of preferences and strategies due to learning effects. Participants were paid in in Singapore dollars.

### Experimental design

#### Uncertainty preference tasks

Uncertainty preference (risk and ambiguity) and choice strategy were evaluated using our gains choice task and losses choice task (Stanton et al., 2011; Kurnianingsih et al., 2015; Mullette-Gillman et al., 2015). Participants performed these two monetary decision tasks, one featuring choices between possible monetary gains and the other between possible monetary losses. On each trial, participants chose between a certain option and a gamble option with varied value and probability of winning (Figure 1B). Data collection and analyses were achieved using MATLAB (Mathworks, Natick, MA) with Psychtoolbox (www.psychtoolbox.org)

The gains choice task consisted of 165 trials (see full description in Stanton et al., 2011; Kurnianingsih et al., 2015; Mullette-Gillman et al., 2015 see full trial metrics in Table S1). On each trial, the participants chose between a certain monetary option (such as $3) and a gamble that was either risky or ambiguous. The 135 risk trials contained a gamble with a known probability of winning a presented value (such as 50% of $8) against a fixed alternative of receiving $0. The 30 ambiguity trials had the same form, except that the gamble had an unknown probability of winning. Risk and ambiguity trials were intermixed and randomized across participants. The matrix of presented risky gambles was constructed from 5 certain gain options {$3, $4, $5, $6, $7}, three probabilities of winning {25%, 50%, 75%}, and nine different relative expected values (rEV or the ratio of the expected value of the gamble to the value of the certain option, EV_G_/V_c_) which were {0.5, 1.0, 1.3, 1.6, 1.9, 2.2, 2.5, 3.0, 3.5}. Potential win values ranged from $2 to $98. For ambiguous gambles, six rEVs were examined {0.5, 1.0, 2.0, 3.0, 4.0, 6.0}, and presented gamble values were calculated based on an assumed 50% probability of winning, resulting in values ranging from $3 to $84. For both risky and ambiguous options, a loss would always result in $0 outcome. Gamble values were rounded to the nearest 10 cents for presentation during choices.

The losses choice task consisted of 200 trials, with 150 risk trials and 50 ambiguity trials. The losses choice task was based on the gains choice task, with altered valence and adjusted rEV values. The adjusted set of 10 rEVs {0.1, 0.3, 0.5, 0.8, 1.0, 1.3, 1.5, 2.0, 3.0, 4.0} were utilized across both risk and ambiguity trials, resulting in potential losses ranging from -$0.40 to -$112.

#### Quantifying uncertainty preferences

**Figure 2:**
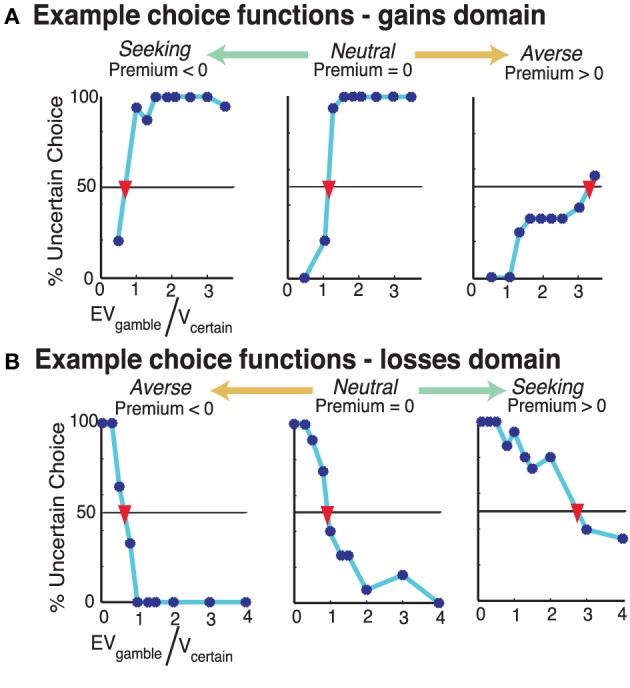
**Examples choice functions for individual participants**. Each subplot shows the choice behavior for 1 participant across the trials where they chose between a risky gamble and a certain monetary option one task. The y-axis indicates the percent at which they selected the gamble option, across 15 trials for each point. The x-axis is the ratio of the expected value of the gamble to the value of the certain option (rEV). **(A)** In the gains choice task, for 135 risk trials across 9 examined rEV values. **(B)** In the losses choice task, for 150 risk trials across 10 examined rEV values. Participants' choice functions for the risk trials from each domain-specific task were plotted to show the percent at which they selected the gamble for each examined rEV value. The indifference point of each participant was determined as the projected rEV for where their choice function indicated 50% selection of the gamble (as indicated with an inverted red triangle for these participants). We defined the risk premium value for each participant as their indifference point −1 (to make zero be risk neutral).

We quantified uncertainty preferences using psychometric indifference point analyses to identify the rEV at which the participant would choose the gamble option 50% of the time, thus indicating indifference between the certain and gamble options. Choice functions were constructed by plotting a continuous function based upon the percentage of accepting the gamble option (y-axis) to each respective assigned rEV (EV_G_/V_c_). Examples of choice functions for both domains are shown in Figure 2. The choice functions were generally monotonic and the first point where the percentage of accepting the gamble option crossed 50% was defined to be the indifference point. We determined the premium value by subtracting 1 from this indifference point to produce a premium value metric with zero indicative of risk neutrality. These analyses were conducted separately for risk and ambiguity and across gains and losses, resulting in four independent uncertainty premium values for each participant.

The premium value is a measure of the degree to which participant alters the valuation of the absolute expected value of the gamble in relation to outcome uncertainty. As such, a zero premium value reflects no change in valuation [subjective value (SV) = expected value (EV)], a positive value indicates diminished valuation (SV < EV) and a negative value indicates enhanced valuation (SV > EV). This interpretation of valuation applies for both gains and losses premium values. However, in the gains domain, positive/negative premium values indicate risk aversion/seeking. But in the losses domain negative/positive premium values indicate risk aversion/seeking. In both domains, a zero risk premium value indicates risk neutrality.

Our premium metric cannot be derived for participants whose choice functions do not have an indifference point within our sampled range (their choice function does not cross 50% acceptance, see Figure 2). We opted to remove such participants from uncertainty preference analyses, as such choice functions are the result of participants not modulating their choice behavior across our large set of relative values of the gamble and certain options (for discussion, see Kurnianingsih et al., 2015). This resulted in the exclusion of: risk gains: 7 RW and 6 TSD; risk losses: 1 RW and 1 TSD; ambiguity gains: 11 RW and 10 TSD; and ambiguity losses: 1 RW and 1 TSD. We note that the exclusion rates across RW and TSD sessions are almost identical.

To facilitate comparison across studies, we note that our risk premium formulation has been previously shown to result in very high correlations with the power function risk preference values, when examined in a large sample (N~300, r > |0.6|for gains, Stanton et al., 2011) or two moderate samples (*N* = 62, *r* > |0.7| for gains and losses, Kurnianingsih et al., 2015; *N* = 72, *r* > |0.49| for gains, and *r* > |0.77| for losses, Mullette-Gillman et al., 2015). In the current smaller sample, in RW, we find slightly lower correlations for risk preferences in gains [*r*_(20)_ = −0.41, *p* = 0.06] and very high correlations in losses [*r*_(26)_ = −0.87, *p* < 0.0001]. For ambiguity preferences, we find uniformly high correlations [gains, *r*_(16)_ = −0.81, *p* < 0.001; losses, *r*_(26)_ = −0.90, *p* < 0.0001], in concurrence with our prior results (*N* = 72, *r* > |0.77| for gains, and *r* > |0.87| for losses, Mullette-Gillman et al., 2015). These consistently high correlations indicate that these two measures of risk preferences are largely capturing the same variance across participants.

#### Quantifying choice strategy

Choice strategy refers to the relative use of two conflicting information types present in each trial that the participant can rely upon to make their choice—the first is the relative expected value of the two options (rEV), and the second is the probability of winning the gamble (pWIN) (see Kurnianingsih et al., 2015; Mullette-Gillman et al., 2015). While rEV is more likely to lead to higher average rewards, it requires multiple steps to calculate. In contrast, while pWIN information is readily available perceptually, it will lead to lower average rewards. Importantly, across the risk trials of our task, the pWIN and rEV of each trial are fully dissociable (their correlation is zero).

To quantify the choice strategy of each participant, for each domain (gains or losses) we utilized two linear regressions (2 factors × 2 domains) (Kurnianingsih et al., 2015; Mullette-Gillman et al., 2015). Each of these four linear regressions determined the influence of one factor (pWIN or rEV) on the choices of a participant across all risk trials within one domain (gains or losses). The produced R-squared values provide the proportion of choice variance that can be explained by each examined factor (see **Figures 4A–D**). Therefore, high R-squared values indicate that choices were most likely influenced by that trial information (e.g., a participant that determines their choices solely based on the probability of winning the gamble would have a high R-squared value for the pWIN factor, while a participant whose decision is solely based upon the ratio of the expected value of the gamble to the value of the certain option would have a high R-squared value for the rEV factor), whereas low R-squared values indicate that choices were based on other factors or were made randomly.

We produced the Choice Strategy metric by taking the difference between these R-squared values (rEV minus pWIN) for each domain (see Figures **4E–H**). As such, the choice strategy metric directly contrasts utilization of the cognitively demanding calculation of the relative expected values of the certain and the gamble option against utilization of the probability of winning in the gamble option. Choice strategy values are positive when participants utilize rEV information more and negative when they utilize pWIN information more. Participants are considered to be “maximizing” when they have positive choice strategy (R-squared rEV > R-squared pWIN) and “satisficing” when they have negative choice strategy (R-squared rEV < R-squared pWIN).

In addition to determination of the choice strategy metric, we also utilized the components of these analyses to examine the question of whether TSD results in an overall decrease in information use. To test this, we calculated a total strategy metric for each participant (for each domain and in each state), as the sum of the R-squared values for the rEV and pWIN regressions. It is important to note that these trial factors are fully orthogonal across trials (i.e., the correlation across trials is zero), so it is mathematically impossible for these factors to account for the same variability in choice behavior in their independent regressions.

We note that as our analytic method only examines the influences of the pWIN and rEV information on the choice behaviors of each participant, as proxies for satisficing and maximizing strategies, we are unable to speak to whether sleep deprivation may have differential effects on the use of other unexamined informational factors. However, we note specifically that one additional factor is very highly correlated with the rEV factor in both tasks—the difference in expected value of the gamble option and the value of the certain option (EVdiff = EV_g_ – V_c_), Across trials, this factor has an extremely high inter-trial correlation with the rEV informational factor [gains: *r*_(133)_ = 0.926, *p* < 0.0001; losses: *r*_(148)_ = 0.957, *p* < 0.0001], and is almost orthogonal to the pWIN factor [gains: *r*_(133)_ < 0.0001, *p* > 0.99; losses: *r*_(148)_ < 0.0001, *p* > 0.99].

#### Loss aversion task

We quantified loss aversion, or the relative influence of potential losses to potential gains on choice behavior, utilizing a modified version of the task and analyses of Tom and colleagues (Tom et al., 2007). The sole alteration in our version was a reduction in the number of trials, to 64 trials (halving the number of sampled increments in each domain). In brief, participants were first endowed with $20 and, on each trial, were given a choice to keep their endowment or risk part of it on an offered gamble. Gambles always had a 50% chance of a gain and a 50% chance of a loss, with gains ranging from $12 to $40 in increments of $4, while losses ranged from -$6 to -$20 in decrements of -$2 (Figure 1C). Participants indicated whether they accepted each proffered gamble with options of “strongly accept,” “weakly accept,” “weakly reject,” or “strongly reject.” No feedback was provided during the task, to prevent learning effects, and participants were informed in the preliminary session that their final payment would be based upon random selection and resolution of one trial from each iteration of the task at the conclusion of the final session.

#### Quantifying loss aversion

Loss aversion preferences were quantified following the analyses of Tom et al. (2007). In brief, a logistic regression was fit to the choices of each participant to determine the differential influence (beta weights) of the potential loss and gain values of the offered gambles on choice behavior (accept or reject). The ratio of these beta weights produced the loss aversion (λ) metric for each participant. Loss aversion values greater than 1 indicate that an individual's choices were more strongly influenced by the value of the potential losses than the potential gains, while values below 1 indicate the reverse, and values of 1 indicate equal weighting. We collected data from 21 participants. We excluded 4 participants whose behavior in either session indicated unreasonable loss aversion values (such as a lambda of 38). These can be the result of the participant ignoring the value of the possible gain—such as employing a fixed response of accepting all gambles whose loss is below a threshold. Such strategies result in strikingly high loss aversion values, and are not well captured by the theoretical concept of loss aversion. This resulted in a final sample of 17 participants for our loss aversion analyses.

#### Statistical analyses

All comparisons between TSD and RW conditions are performed utilizing within-subject statistical tests.

## Results

### Relationship of economic task metrics

As a baseline, we examined the inter-relationships of our economic metrics within the RW state. Risk and ambiguity premiums were highly correlated within each domain [gains: *r*_(15)_ = 0.86, *p* < 0.0001; losses: *r*_(26)_ = 0.84, *p* < 0.0001]. Risk preferences were uncorrelated with choice strategy within either domain [gains: *r*_(19)_ = 0.17, *p* = 0.46.; losses: *r*_(26)_ = −0.12, *p* = 0.53]. These results concur with our prior results using these tasks in young adults (Kurnianingsih et al., 2015).

We found no significant correlation (all *p* > .05, uncorrected) between loss aversion and uncertainty preferences (risk and ambiguity) or choice strategies within either domain. As loss aversion is the ratio of the relative weighting of losses to gains, we examined for potential relationships between loss aversion and the ratio of risk premiums and choice strategies across domains (losses/gains). No significant correlations were found (all *p* > 0.05, uncorrected), indicating that loss aversion is an independent measure of choice behavior from risk preferences and choice strategy.

In summary, we show very high correlations between risk and ambiguity preferences within a domain, and found no other significant correlations between our decision-making metrics within either domain.

### Sleep deprivation reduces vigilant attention

To confirm the robustness of our TSD manipulation, we examined for alterations of PVT response times and attentional lapses, as impaired vigilance is among the most robust effects of sleep deprivation (Lim and Dinges, 2010). PVT data were available for a subset of 18 participants. On average, participants showed slower median reaction times in TSD than in RW [mean ± SD, RW: 242.8 ± 22.3 ms, TSD: 300.9 ± 50.1 ms; *t*_(13)_ = 6.41, *p* < 0.0001, Cohen's *d* = 1.54]. There was also an increase in the number of attentional lapses under TSD [mean ± SD RW: 0.56 ± 0.78; TSD: 7.67 ± 6.74; *t*_(13)_ = 4.16, *p* < 0.0001, Cohen's *d* = 1.52].

### Sleep deprivation does not alter the response times of economic decisions

Given that TSD alters PVT response times, we examined whether TSD also alters response times for economic decisions (Table 1). We found no significant effects of sleep deprivation on response times within either the gains [mean ± SD risk difference: 0.002 ± 0.23; *t*_(28)_ = 0.06, *p* = 0.95, Cohen's *d* = 0.008; mean ± SD ambiguity difference: −0.02 ± 0.26; *t*_(28)_ = 0.48, *p* = 0.63, Cohen's *d* = 0.07] or the losses domains [rmean ± SD risk difference: −0.04 ± 0.20; *t*_(28)_ = 0.97, *p* = 0.34, Cohen's *d* = 0.12; mean ± SD ambiguity difference: −0.03 ± 0.24; *t*_(28)_ = 0.62, *p* = 0.54, Cohen's *d* = 0.10]. Within both states, participants exhibited faster response times for the gains choice task than the losses choice task [risk RW: *t*_(28)_ = 3.52, *p* < 0.003, Cohen's *d* = 0.33; TSD: *t*_(28)_ = 2.16, *p* < 0.039, Cohen's *d* = 0.38; ambiguity RW: *t*_(28)_ = 2.50, *p* = 0.02, Cohen's *d* = 0.46; TSD: *t*_(28)_ = 1.73, *p* = 0.09, Cohen's *d* = 0.32].

**Table 1:** **Comparison of economic measures between RW and TSD**.

	**Rested Wakefulness (RW) mean ± SD**	**Sleep Deprivation (TSD) mean ± SD**	*RW vs. TSD p*-value^***^
**GAINS CHOICE TASK**			
**Uncertainty premiums**			
Risk (22, 23)^*^	0.48 ± 0.68	0.52 ± 0.78	0.73
Ambiguity (18, 19)	1.52 ± 1.37	1.28 ± 1.46	0.76
**Strategy**			
Choice strategy (29, 29)	−0.007 ± 0.27	−0.09 ± 0.30	**0.01**
rEV r^2^ (29, 29)	0.19 ± 0.15	0.15 ± 0.14	**0.015**
pWIN r^2^ (29, 29)	0.19 ± 0.17	0.23 ± 0.19	**0.031**
Total strategy (29, 29)	0.38 ± 0.14	0.38 ± 0.15	0.97
Response time (s)^**^			
Risk (29, 29)	1.02 ± 0.31	1.01 ± 0.34	0.95
Ambiguity (29, 29)	1.04 ± 0.35	1.06 ± 0.37	0.63
**LOSSES CHOICE TASK**			
**Uncertainty premiums**			
Risk (28, 28)	0.01 ± 0.43	−0.03 ± 0.29	0.40
Ambiguity (28, 28)	0.01 ± 0.50	−0.10 ± 0.38	0.07
**Strategy**			
Choice strategy (29, 29)	0.36 ± 0.15	0.34 ± 0.13	0.32
rEV r^2^ (29, 29)	0.40 ± 0.12	0.38 ± 0.11	0.40
pWIN r^2^ (29, 29)	0.04 ± 0.04	0.04 ± 0.05	0.42
Total strategy (29, 29)	0.43 ± 0.10	0.42 ± 0.12	0.57
Response time (s)^**^			
Risk (29, 29)	1.14 ± 0.30	1.10 ± 0.29	0.34
Ambiguity (29, 29)	1.19 ± 0.32	1.16 ± 0.27	0.54
**LOSS AVERSION TASK**			
Loss aversion (17, 17)	1.98 ± 1.40	2.16 ± 1.87	0.30

* *Numbers in parentheses indicate the number of subjects in each group (N RW, N TSD)*.

** *Median response time*.

*** *Paired-sample t-tests*.

*Abbreviations: rEV, Relative Expected Value; pWIN, Probability of Winning; s, seconds*.

*Bolded values indicate statistical significance at p < 0.05*.

### Sleep deprivation does not alter risk or ambiguity preferences

To examine the test-retest reliability of our uncertainty preference metrics, we ran a correlation across sessions. With ~1 week between sessions, and no resolution of any gambles until the end of all sessions, we found very strong test-retest reliability between uncertainty preferences for all four uncertainty preference measures [correlations; risk gains: *r*_(19)_ = 0.78, *p* < 0.0001; risk losses: *r*_(26)_ = 0.79, *p* < 0.0001; ambiguity gains: *r*_(12)_ = 0.55, *p* = 0.04; ambiguity losses: *r*_(26)_ = 0.81, *p* < 0.0001].

**Figure 3:**
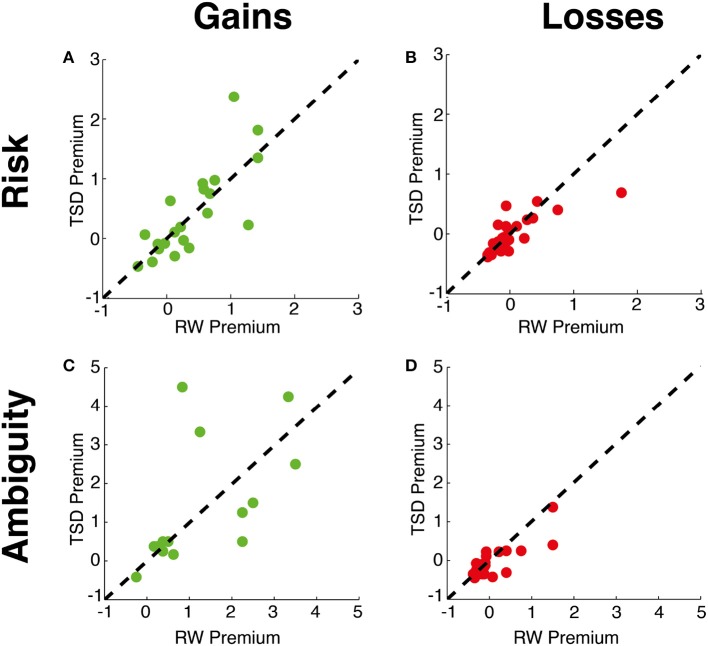
**Uncertainty preferences**. Relationship between subjects' preferences across RW and TSD conditions for **(A)** Gains risk premium, **(B)** Losses risk premium, **(C)** Gains ambiguity premium, and **(D)** Losses ambiguity premium.

Sleep deprivation did not shift risk preferences within either the gains [mean ± SD difference: 0.04 ± 0.47; *t*_(20)_ < 1, *p* = 0.73, Cohen's *d* = 0.06] or the losses domains [mean ± SD difference: −0.04 ± 0.26; *t*_(27)_ < 1, *p* = 0.40, Cohen's *d* = 0.12] (see Table 1 and Figure 3). Risk preferences under both RW and TSD followed the classical pattern (Kahneman and Tversky, 1979), with people generally risk averse for gains and risk neutral or seeking for losses [mean ± SD, Gains RW: 0.48 ± 0.68, TSD: 0.52 ± 0.78; Losses RW: 0.01 ± 0.43, TSD: −0.03 ± 0.29].

There was also no significant difference in ambiguity preferences between RW and TSD within the gains domain [mean ± SD difference: 0.12 ± 1.38; *t*_(13)_ < 1, *p* = 0.76, Cohen's *d* = 0.17]. Within the losses domain, there was a non-significant trend suggesting higher ambiguity seeking during TSD [mean ± SD difference: −0.11 ± 0.29; *t*_(27)_ = 1.90, *p* = 0.061, Cohen's *d* = 0.24], with calculation of Cohen's *d* indicating a small effect size (Cohen, 1988). Overall, participants were significantly more ambiguity averse than risk averse in the gains domain [RW: *t*_(16)_ = 4.71, *p* < 0.0001, Cohen's *d* = 1.03; TSD: *t*_(17)_ = 2.92, *p* = 0.010, Cohen's *d* = 0.69], while no difference between ambiguity and risk preference was found in the losses domain [RW: *t*_(27)_ < 1, *p* = 0.91, Cohen's *d* = 0.01; TSD: *t*_(27)_ = 1.31, *p* = 0.20, Cohen's *d* = 0.21].

### Sleep deprivation does not alter loss aversion preference

We examined the effect of TSD on loss aversion preferences. With ~1 week separation and no resolution of outcomes, loss aversion preferences were highly stationary, with test-retest correlations of [*r*_(15)_ = 0.95, *p* < 0.0001]. Examining for modulation of loss aversion by TSD, we found no significant effect [mean ± SD difference λ: 0.18 ± 0.70, *t*_(16)_ = 1.06, *p* = 0.30, Cohen's *d* = 0.11].

### Choice strategy is highly stationary over sessions

We examined the information that each participant utilized to make their choices through our choice strategy metric. Across sessions, with ~1 week delay and no resolution of outcomes, we found very strong test-retest reliability between choice strategy values within both domains [correlations; gains: *r*_(27)_ = 0.84, *p* < 0.0001; losses: *r*_(27)_ = 0.72, *p* < 0.0001]. This is a strong concurrence in the test-retest reliability of these measures, building upon our previously published 90-min delay (correlations; gains: *r* > 0.89; losses: *r* > 0.77; Mullette-Gillman et al., 2015).

### For gains, sleep deprivation decreases use of maximizing information and increases use of satisficing information

**Figure 4:**
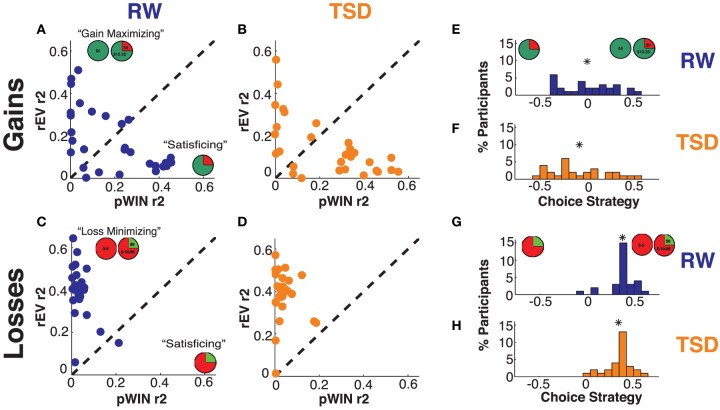
**Choice strategy metric—utilization of trial information**. Relationship between trial variances explained (R-squared) by the rEV and pWIN information for **(A)** RW gains domain, **(B)** TSD gains domain, **(C)** RW losses domain, and **(D)** TSD losses domain. Distribution of choice strategy values (difference between rEV R-squared minus pWIN R-squared) for **(E)** RW gains domain, **(F)** TSD gains domain, **(G)** RW losses domain, and **(H)** TSD losses domain. The “^*^” indicates the mean of each distribution.

**Figure 5:**
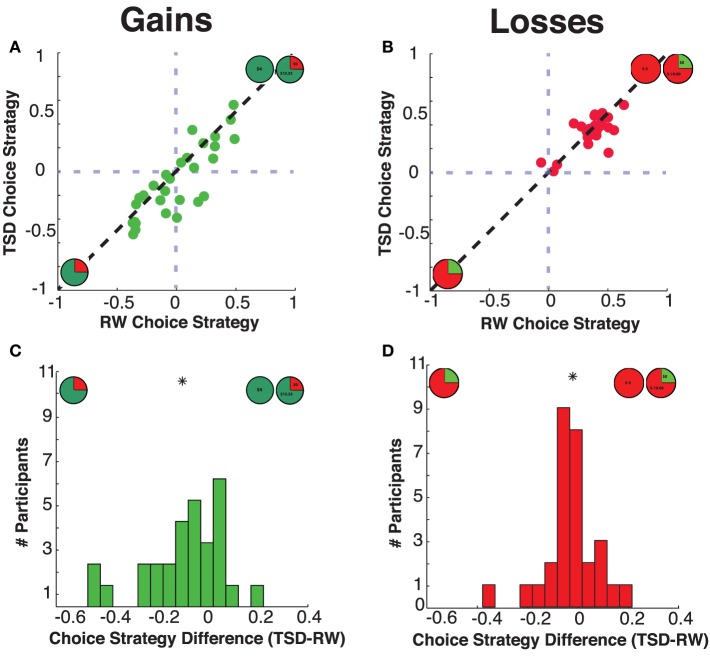
**Choice strategies**. Relationship between subjects' choice strategy across rested wakefulness (RW) and total sleep deprivation (TSD) conditions for **(A)** Gains choice strategy, and **(B)** Losses choice strategy. Distribution of differences in choice strategy (TSD minus RW) for the **(C)** Gains domain and the **(D)** Losses domain. The “^*^” indicates the mean of each distribution.

Within the gains domain, we found significant modulation of choice strategy by sleep deprivation condition [mean ± SD difference: −0.09 ± 0.16, *t*_(28)_ = 2.78, *p* = 0.010 Cohen's *d* = 0.31]. Concurring with our hypotheses, TSD resulted in decreased choice strategy; that is, diminished use of maximizing information relative to satisficing information (see Figures 4, 5). As the choice strategy metric is a composite metric, we examined the effect of TSD on the independent R-squared values for the two component factors—rEV and pWIN. Within the gains domain, TSD resulted in significant alteration of both components—diminished use of the maximizing rEV information [mean ± SD difference: −0.04 ± 0.09, *t*_(28)_ = 2.58, *p* = 0.015, Cohen's *d* = 0.31] and increased use of the satisficing pWIN information [mean ± SD difference: 0.05 ± 0.11, *t*_(28)_ = 2.27, *p* = 0.031, Cohen's *d* = 0.25].

Given this clear decrease in strategy in the gains domain, we examined if this change in behavior was related to oft-found reductions in vigilance. Within the 18 participants with vigilance data, we found that individual differences in the effects of TSD on reaction times (PVT RT, TSD-RW) were significantly negatively correlated with the effects of TSD on choice strategy (TSD-RW) in the gains domains [*r*_(16)_ = −0.47, *p* = 0.047]—individuals in which TSD led to longer reaction times in the psychomotor vigilance task also showed reduced choice strategy in the gains choice task.

### For losses, sleep deprivation does not alter information use

Within the losses domain, we did not find a significant mean shift in choice strategy between RW and TSD [mean ± SD difference: −0.02 ± 0.10, *t*_(28)_ = 1.01, *p* = 0.32, Cohen's *d* = 0.14].

### Sleep deprivation does not alter total information use

To test whether TSD resulted in a decrease in the total information that participants used to make their economic choices, we examined our total strategy metric (the sum of the R-squared values of the rEV and pWIN regressions). The total strategy metric showed high within-subject stationarity across states within both domains [gains, *r*_(27)_ = 0.72, *p* < 0.0001; losses, *r*_(27)_ = 0.67, *p* < 0.0001]. Testing for TSD effects, we found no significant differences between RW and TSD within either the gains or losses domains [mean ± SD gains difference: 0.005 ± 0.11, *t*_(28)_ = 0.04, *p* = 0.81, Cohen's *d* = 0.03; losses difference: −0.01 ± 0.09, *t*_(28)_ = 0.57, *p* = 0.57, Cohen's *d* = 0.09).

### Replicating information use analyses with logistic regressions

The choice patterns of subjects range from linear to logistic across the rEV values (as can be seen in Figure 2). Use of linear models could potentially produce misestimations in participants whose choice functions are more logistic (specifically, a participant with a sharp transition will have a lower R-squared value than a matched participant with a gentle slope). Nonetheless, we chose to use linear models due to a number of competing items: (1) answering our hypotheses required two independent regressions for each participant, to determine the independent contributions (R-squared values) of the pWIN and rEV trial factors; (2) with linear models, the cross-trial correlation of zero between the pWIN and rEV factors prevents omitted-factor bias (i.e., the misestimation of the contribution of a factor due to the absence of the second factor in the regression); and (3) with independent logistic models, omitted-factor bias would be present regardless of the correlation between the included and excluded factors. Although our use of linear models is imperfect, the impact on our principle findings is likely negligible (Hellevik, 2009).

Critically, any such misestimations are independent of the manipulation that is the focus of this manuscript. In each condition (RW or TSD), reduced precision of the degree to which a participant utilized the rEV factor would result in on-average symmetric noise of the point-estimation (reducing overall power), but without a directional bias on the examined manipulation effects (as there is no relationship to the manipulation). We note that the high individual test-retest stationarity we reported previously (Section Choice Strategy is Highly Stationary Over Sessions, with correlations gains *r* = 0.84 and losses *r* = 0.72) suggests that the cumulative effects of any such imprecisions are small. In fact (below), we find the exact same pattern of manipulation effects using logistic models as was previously found using linear models.

To test the analytic robustness of our found patterns of change in information use, we replicated our analyses replacing the linear regressions with logistic regressions, and calculating McFadden's pseudo R-squared (McFadden, 1974). We found very high correlations between the R-squared and pseudo R-squared values from the linear and logistic regressions across the RW and TSD sessions, within both the gains [rEV: *r*_(56)_ = 0.90, *p* < 0.0001; pWIN: *r*_(56)_ = 0.90, *p* < 0.0001] and losses [rEV: *r*_(56)_ = 0.73, *p* < 0.0001; pWIN: *r*_(56)_ = 0.99, *p* < 0.0001] domains. We replicated all results contrasting the RW and TSD sessions—a significant decrease in the use of rEV information and an increase in pWIN information in the gains domain only [rEV: gains, *t*_(28)_ = 2.95, *p* = 0.006, Cohen's *d* = 0.29; losses, *t*_(28)_ = 1.45, *p* = 0.16, Cohen's *d* = 0.23; pWIN: gains, *t*_(28)_ = 2.15, *p* = 0.041, Cohen's *d* = 0.24; losses, *t*_(28)_ = 0.46, *p* = 0.65, Cohen's *d* = 0.06], and no alteration of total information used in either domain [the sum of rEV and pWIN pseudo R-squared values; gains, *t*_(28)_ = 0.59, *p* = 0.56, Cohen's *d* = 0.09; losses, *t*_(28)_ = 1.36, *p* = 0.18, Cohen's *d* = 0.24].

We note that we could not simply run a multi-factor regression, as it would provide only a single R-squared value (accounting for the joint variance accounted for by both the pWIN and rEV factors) and the estimated coefficients definitionally address a different dimension of behavior (the directional influence of the factor, as opposed to the amount of variance it can explain).

## Discussion

We show that sleep deprivation alters economic decision making through alterations of choice strategies. TSD did not significantly alter economic preferences (risk, ambiguity, or loss aversion), decision response times, or the total information used by participants. In contrast, we found that one night of sleep deprivation altered the information that participants relied upon to make their choices, specifically within the gains domain.

In the gains domain, TSD produced a general decrease in choice strategy, which was the result of both decreased use of maximizing information (rEV) and increased use of satisficing information (pWIN). TSD did not alter the total amount that participants utilized these types of information, indicating that in economic decision making TSD produces a switch in what information participants rely upon rather than a decrease in overall information use.

### TSD does not alter uncertainty preferences

Our participants exhibited the standard pattern of average uncertainty preferences across the gains and losses domains, during both RW and TSD; that is, participants were, on average, risk averse for gains and risk seeking/neutral for losses (Kahneman and Tversky, 1979). With high test-retest reliability, we found no alterations in uncertainty preferences in either the gains or the losses domains. This represents the first study to explicitly test sleep deprivation effects on uncertainty preferences while controlling for potentially confounding factors such as strategy and learning.

We note that, as compared to our findings, several prior studies have suggested that TSD results in altered uncertainty preferences. This discrepancy may be due to task and metric differences, with previous studies unable to dissociate alterations in uncertainty preferences from related cognitive processes such as reward learning (as in the Iowa Gambling Task, Killgore et al., 2006). Beyond explicitly testing uncertainty preferences, our task design also allowed us to distinguish alterations of preferences from alterations of strategies. We suggest that this conflict can be resolved through consideration of our observed alterations of choice strategies (see below). That is, prior studies may have ascribed behavioral alterations to preference shifts that may actually have been due to changes in choice strategy.

### TSD does not alter loss aversion preferences

We also examined whether TSD results in a change in loss aversion, or the relative weighting of losses and gains. We found no alteration of loss aversion preferences, concurring with the behavioral findings of Venkatraman et al. (2011) and their suggestion that behavioral alterations are due to other factors.

### TSD decreases choice strategy in the gains domain

Within the gains domain, TSD resulted in a significant decrease in our choice strategy metric. As this is a compound metric, we examined the components and found that TSD both decreased the use of the relative expected value information (rEV) and increased the use of probability information (pWIN). Use of the rEV may be considered a form of “maximizing strategy” (maximizes expected outcomes, but requires multiple cognitive steps to calculate), while use of pWIN may be considered a “satisficing strategy” (a simplifying heuristic that utilizes readily-available information at the cost of maximizing rewards). As such, in the gains domain TSD led to decreased use of maximizing strategies and concomitant increased use of satisficing strategies. This result concurs with a recent study by Menz et al. (2012), which found a reduction in decision-making quality (higher stochasticity) without a change in preferences.

Further, the individual change (TSD-RW) in choice strategy in the gains domain showed a negative correlation with change in performance on the psychomotor vigilance task. Given the inversion between these scales, with poorer performance corresponding to higher PVT response times and lower choice strategy values, this relationship shows that the individuals who had the most detrimental effect of TSD modulation on PVT also had the greatest reduction in maximizing behavior. This relationship suggests that the mechanisms through which sleep deprivation alters choice strategy in the gains domain is related to the mechanisms for altered response times in the PVT.

### Inferring cognitive alterations

Does this pattern of alterations allow us to determine what cognitive processes are affected by sleep deprivation? TSD specifically altered strategy in the gains domain, with decreased use of maximizing information and increased use of satisficing information. Notably, there was a correlation between the degree of change (TSD-RW) in choice strategy and the change in psychomotor vigilance. However, TSD did not alter uncertainty preferences (risk or ambiguity) or loss aversion. These results clearly show the independence of effects in the gains and losses domains, suggesting that the cognitive or neural mechanisms are not simple mirrors. In addition, the domain-specificity of our alteration suggests a possible alternate explanation for the TSD-produced optimism bias found by Venkatraman et al. (2011). In brief, a gains-specific increase in satisficing behavior could have biased behavior in their 5-outcome mixed gamble task (rather than a change in valuation).

Are these results interpretable through a simple two-system model of affect vs. reason? If TSD specifically alters affective processes, such as the subjective valuation of gains or losses, then we may have seen clear changes in risk preferences in either domain. If TSD alters the relative valuation of gains and losses, then we may have seen clear changes in loss aversion. We saw neither of these.

Rather, the effects of TSD were limited to the strategy measures. Based on a dual-system model, this pattern of changed strategy with unaltered preferences could be interpreted to suggest that TSD alters cognitive processes related to reason without altering affect. However, in disagreement, we also found no reduction in overall information use, and the changes in strategy were limited to the gains domain.

We caution against interpreting these results based upon such a dual-system approach. Simply, changes in strategy could be the result of changes in reason or motivation. Similarly, changes in risk preferences or loss aversion could not only be produced by altered affect, but could also be derived from changes in reasoning alone.

The clearest indication of the altered cognitive processes responsible for our found alteration in gains strategy come from the strong relationship between the change in strategy and the change in psychomotor vigilance (PVT). This relationship suggests that these effects share an underlying source, but unfortunately our results cannot specify that source.

## Conclusions

TSD alters the information participants rely upon to make their decisions, without modulating uncertainty preferences (risk and ambiguity), loss aversion, or decision time. In gains, we identified a decrease in use of maximizing information with a concomitant increase in the use of satisficing information. TSD did not decrease the overall information use.

These results clearly indicate that sleep deprivation negatively impacts decision making in the gains domains, which will lead to lost gains. Such specification of the effects of sleep deprivation on human decision making is critical for the production of effective treatments and policy, including interventions and training for individuals who face unavoidable sleep deprivation (e.g., due to career, medical conditions, or parenting).

## Author contributions

OM designed the study. JL coordinated data collection. OM and YK analyzed and interpreted the data. OM and YK wrote the manuscript with comments from JL.

### Conflict of interest statement

The authors declare that the research was conducted in the absence of any commercial or financial relationships that could be construed as a potential conflict of interest.
