# Supplementary material for: Sleep deprivation alters choice strategy without altering uncertainty or loss aversion preferences
Source: Front Neurosci. 2015 Oct 6;9:352. doi: 10.3389/fnins.2015.00352 (PMC4593856; doi:10.3389/fnins.2015.00352)
Supplement: Supplementary file 1 [file Table1.DOCX]

Table S1. Option pairs presented in the risk task

| **Gains Risk Trials** | | | | |
| --- | --- | --- | --- | --- |
| Trial # | Certain Value ($) | pWIN  (%) | Gamble Value ($) | rEV |
| 1 | 3 | 0.25 | 6 | 0.5 |
| 2 | 3 | 0.5 | 3 | 0.5 |
| 3 | 3 | 0.75 | 2 | 0.5 |
| 4 | 3 | 0.25 | 12 | 1 |
| 5 | 3 | 0.5 | 6 | 1 |
| 6 | 3 | 0.75 | 4 | 1 |
| 7 | 3 | 0.25 | 15.6 | 1.3 |
| 8 | 3 | 0.5 | 7.8 | 1.3 |
| 9 | 3 | 0.75 | 5.2 | 1.3 |
| 10 | 3 | 0.25 | 19.2 | 1.6 |
| 11 | 3 | 0.5 | 9.6 | 1.6 |
| 12 | 3 | 0.75 | 6.4 | 1.6 |
| 13 | 3 | 0.25 | 22.8 | 1.9 |
| 14 | 3 | 0.5 | 11.4 | 1.9 |
| 15 | 3 | 0.75 | 7.6 | 1.9 |
| 16 | 3 | 0.25 | 26.4 | 2.2 |
| 17 | 3 | 0.5 | 13.2 | 2.2 |
| 18 | 3 | 0.75 | 8.8 | 2.2 |
| 19 | 3 | 0.25 | 30 | 2.5 |
| 20 | 3 | 0.5 | 15 | 2.5 |
| 21 | 3 | 0.75 | 10 | 2.5 |
| 22 | 3 | 0.25 | 36 | 3 |
| 23 | 3 | 0.5 | 18 | 3 |
| 24 | 3 | 0.75 | 12 | 3 |
| 25 | 3 | 0.25 | 42 | 3.5 |
| 26 | 3 | 0.5 | 21 | 3.5 |
| 27 | 3 | 0.75 | 14 | 3.5 |
| 28 | 4 | 0.25 | 8 | 0.5 |
| 29 | 4 | 0.5 | 4 | 0.5 |
| 30 | 4 | 0.75 | 2.7 | 0.5 |
| 31 | 4 | 0.25 | 16 | 1 |
| 32 | 4 | 0.5 | 8 | 1 |
| 33 | 4 | 0.75 | 5.3 | 1 |
| 34 | 4 | 0.25 | 20.8 | 1.3 |
| 35 | 4 | 0.5 | 10.4 | 1.3 |
| 36 | 4 | 0.75 | 6.9 | 1.3 |
| 37 | 4 | 0.25 | 25.6 | 1.6 |
| 38 | 4 | 0.5 | 12.8 | 1.6 |
| 39 | 4 | 0.75 | 8.5 | 1.6 |
| 40 | 4 | 0.25 | 30.4 | 1.9 |
| 41 | 4 | 0.5 | 15.2 | 1.9 |
| 42 | 4 | 0.75 | 10.1 | 1.9 |
| 43 | 4 | 0.25 | 35.2 | 2.2 |
| 44 | 4 | 0.5 | 17.6 | 2.2 |
| 45 | 4 | 0.75 | 11.7 | 2.2 |
| 46 | 4 | 0.25 | 40 | 2.5 |
| 47 | 4 | 0.5 | 20 | 2.5 |
| 48 | 4 | 0.75 | 13.3 | 2.5 |
| 49 | 4 | 0.25 | 48 | 3 |
| 50 | 4 | 0.5 | 24 | 3 |
| 51 | 4 | 0.75 | 16 | 3 |
| 52 | 4 | 0.25 | 56 | 3.5 |
| 53 | 4 | 0.5 | 28 | 3.5 |
| 54 | 4 | 0.75 | 18.7 | 3.5 |
| 55 | 5 | 0.25 | 10 | 0.5 |
| 56 | 5 | 0.5 | 5 | 0.5 |
| 57 | 5 | 0.75 | 3.3 | 0.5 |
| 58 | 5 | 0.25 | 20 | 1 |
| 59 | 5 | 0.5 | 10 | 1 |
| 60 | 5 | 0.75 | 6.7 | 1 |
| 61 | 5 | 0.25 | 26 | 1.3 |
| 62 | 5 | 0.5 | 13 | 1.3 |
| 63 | 5 | 0.75 | 8.7 | 1.3 |
| 64 | 5 | 0.25 | 32 | 1.6 |
| 65 | 5 | 0.5 | 16 | 1.6 |
| 66 | 5 | 0.75 | 10.7 | 1.6 |
| 67 | 5 | 0.25 | 38 | 1.9 |
| 68 | 5 | 0.5 | 19 | 1.9 |
| 69 | 5 | 0.75 | 12.7 | 1.9 |
| 70 | 5 | 0.25 | 44 | 2.2 |
| 71 | 5 | 0.5 | 22 | 2.2 |
| 72 | 5 | 0.75 | 14.7 | 2.2 |
| 73 | 5 | 0.25 | 50 | 2.5 |
| 74 | 5 | 0.5 | 25 | 2.5 |
| 75 | 5 | 0.75 | 16.7 | 2.5 |
| 76 | 5 | 0.25 | 60 | 3 |
| 77 | 5 | 0.5 | 30 | 3 |
| 78 | 5 | 0.75 | 20 | 3 |
| 79 | 5 | 0.25 | 70 | 3.5 |
| 80 | 5 | 0.5 | 35 | 3.5 |
| 81 | 5 | 0.75 | 23.3 | 3.5 |
| 82 | 6 | 0.25 | 12 | 0.5 |
| 83 | 6 | 0.5 | 6 | 0.5 |
| 84 | 6 | 0.75 | 4 | 0.5 |
| 85 | 6 | 0.25 | 24 | 1 |
| 86 | 6 | 0.5 | 12 | 1 |
| 87 | 6 | 0.75 | 8 | 1 |
| 88 | 6 | 0.25 | 31.2 | 1.3 |
| 89 | 6 | 0.5 | 15.6 | 1.3 |
| 90 | 6 | 0.75 | 10.4 | 1.3 |
| 91 | 6 | 0.25 | 38.4 | 1.6 |
| 92 | 6 | 0.5 | 19.2 | 1.6 |
| 93 | 6 | 0.75 | 12.8 | 1.6 |
| 94 | 6 | 0.25 | 45.6 | 1.9 |
| 95 | 6 | 0.5 | 22.8 | 1.9 |
| 96 | 6 | 0.75 | 15.2 | 1.9 |
| 97 | 6 | 0.25 | 52.8 | 2.2 |
| 98 | 6 | 0.5 | 26.4 | 2.2 |
| 99 | 6 | 0.75 | 17.6 | 2.2 |
| 100 | 6 | 0.25 | 60 | 2.5 |
| 101 | 6 | 0.5 | 30 | 2.5 |
| 102 | 6 | 0.75 | 20 | 2.5 |
| 103 | 6 | 0.25 | 72 | 3 |
| 104 | 6 | 0.5 | 36 | 3 |
| 105 | 6 | 0.75 | 24 | 3 |
| 106 | 6 | 0.25 | 84 | 3.5 |
| 107 | 6 | 0.5 | 42 | 3.5 |
| 108 | 6 | 0.75 | 28 | 3.5 |
| 109 | 7 | 0.25 | 14 | 0.5 |
| 110 | 7 | 0.5 | 7 | 0.5 |
| 111 | 7 | 0.75 | 4.7 | 0.5 |
| 112 | 7 | 0.25 | 28 | 1 |
| 113 | 7 | 0.5 | 14 | 1 |
| 114 | 7 | 0.75 | 9.3 | 1 |
| 115 | 7 | 0.25 | 36.4 | 1.3 |
| 116 | 7 | 0.5 | 18.2 | 1.3 |
| 117 | 7 | 0.75 | 12.1 | 1.3 |
| 118 | 7 | 0.25 | 44.8 | 1.6 |
| 119 | 7 | 0.5 | 22.4 | 1.6 |
| 120 | 7 | 0.75 | 14.9 | 1.6 |
| 121 | 7 | 0.25 | 53.2 | 1.9 |
| 122 | 7 | 0.5 | 26.6 | 1.9 |
| 123 | 7 | 0.75 | 17.7 | 1.9 |
| 124 | 7 | 0.25 | 61.6 | 2.2 |
| 125 | 7 | 0.5 | 30.8 | 2.2 |
| 126 | 7 | 0.75 | 20.5 | 2.2 |
| 127 | 7 | 0.25 | 70 | 2.5 |
| 128 | 7 | 0.5 | 35 | 2.5 |
| 129 | 7 | 0.75 | 23.3 | 2.5 |
| 130 | 7 | 0.25 | 84 | 3 |
| 131 | 7 | 0.5 | 42 | 3 |
| 132 | 7 | 0.75 | 28 | 3 |
| 133 | 7 | 0.25 | 98 | 3.5 |
| 134 | 7 | 0.5 | 49 | 3.5 |
| 135 | 7 | 0.75 | 32.7 | 3.5 |
|  |  |  |  |  |
| **Gains Ambiguity Trials** | | | | |
| Trial # | Certain Value  ($) | pWIN  (%) | Gamble Value  ($) | rEV |
| 1 | 3 |  | 3 | 0.5 |
| 2 | 3 |  | 6 | 1 |
| 3 | 3 |  | 12 | 2 |
| 4 | 3 |  | 18 | 3 |
| 5 | 3 |  | 24 | 4 |
| 6 | 3 |  | 36 | 6 |
| 7 | 4 |  | 4 | 0.5 |
| 8 | 4 |  | 8 | 1 |
| 9 | 4 |  | 16 | 2 |
| 10 | 4 |  | 24 | 3 |
| 11 | 4 |  | 32 | 4 |
| 12 | 4 |  | 48 | 6 |
| 13 | 5 |  | 5 | 0.5 |
| 14 | 5 |  | 10 | 1 |
| 15 | 5 |  | 20 | 2 |
| 16 | 5 |  | 30 | 3 |
| 17 | 5 |  | 40 | 4 |
| 18 | 5 |  | 60 | 6 |
| 19 | 6 |  | 6 | 0.5 |
| 20 | 6 |  | 12 | 1 |
| 21 | 6 |  | 24 | 2 |
| 22 | 6 |  | 36 | 3 |
| 23 | 6 |  | 48 | 4 |
| 24 | 6 |  | 72 | 6 |
| 25 | 7 |  | 7 | 0.5 |
| 26 | 7 |  | 14 | 1 |
| 27 | 7 |  | 28 | 2 |
| 28 | 7 |  | 42 | 3 |
| 29 | 7 |  | 56 | 4 |
| 30 | 7 |  | 84 | 6 |
|  |  |  |  |  |
| **Losses Risk Trials** | | | | |
| Trial # | Certain Value  ($) | pWIN  (%) | Gamble Value  ($) | rEV |
| 1 | -3 | 0.25 | -1.2 | 0.1 |
| 2 | -3 | 0.5 | -0.6 | 0.1 |
| 3 | -3 | 0.75 | -0.4 | 0.1 |
| 4 | -3 | 0.25 | -3.6 | 0.3 |
| 5 | -3 | 0.5 | -1.8 | 0.3 |
| 6 | -3 | 0.75 | -1.2 | 0.3 |
| 7 | -3 | 0.25 | -6 | 0.5 |
| 8 | -3 | 0.5 | -3 | 0.5 |
| 9 | -3 | 0.75 | -2 | 0.5 |
| 10 | -3 | 0.25 | -9.6 | 0.8 |
| 11 | -3 | 0.5 | -4.8 | 0.8 |
| 12 | -3 | 0.75 | -3.2 | 0.8 |
| 13 | -3 | 0.25 | -12 | 1 |
| 14 | -3 | 0.5 | -6 | 1 |
| 15 | -3 | 0.75 | -4 | 1 |
| 16 | -3 | 0.25 | -15.6 | 1.3 |
| 17 | -3 | 0.5 | -7.8 | 1.3 |
| 18 | -3 | 0.75 | -5.2 | 1.3 |
| 19 | -3 | 0.25 | -18 | 1.5 |
| 20 | -3 | 0.5 | -9 | 1.5 |
| 21 | -3 | 0.75 | -6 | 1.5 |
| 22 | -3 | 0.25 | -24 | 2 |
| 23 | -3 | 0.5 | -12 | 2 |
| 24 | -3 | 0.75 | -8 | 2 |
| 25 | -3 | 0.25 | -36 | 3 |
| 26 | -3 | 0.5 | -18 | 3 |
| 27 | -3 | 0.75 | -12 | 3 |
| 28 | -3 | 0.25 | -48 | 4 |
| 29 | -3 | 0.5 | -24 | 4 |
| 30 | -3 | 0.75 | -16 | 4 |
| 31 | -4 | 0.25 | -1.6 | 0.1 |
| 32 | -4 | 0.5 | -0.8 | 0.1 |
| 33 | -4 | 0.75 | -0.5 | 0.1 |
| 34 | -4 | 0.25 | -4.8 | 0.3 |
| 35 | -4 | 0.5 | -2.4 | 0.3 |
| 36 | -4 | 0.75 | -1.6 | 0.3 |
| 37 | -4 | 0.25 | -8 | 0.5 |
| 38 | -4 | 0.5 | -4 | 0.5 |
| 39 | -4 | 0.75 | -2.7 | 0.5 |
| 40 | -4 | 0.25 | -12.8 | 0.8 |
| 41 | -4 | 0.5 | -6.4 | 0.8 |
| 42 | -4 | 0.75 | -4.3 | 0.8 |
| 43 | -4 | 0.25 | -16 | 1 |
| 44 | -4 | 0.5 | -8 | 1 |
| 45 | -4 | 0.75 | -5.3 | 1 |
| 46 | -4 | 0.25 | -20.8 | 1.3 |
| 47 | -4 | 0.5 | -10.4 | 1.3 |
| 48 | -4 | 0.75 | -6.9 | 1.3 |
| 49 | -4 | 0.25 | -24 | 1.5 |
| 50 | -4 | 0.5 | -12 | 1.5 |
| 51 | -4 | 0.75 | -8 | 1.5 |
| 52 | -4 | 0.25 | -32 | 2 |
| 53 | -4 | 0.5 | -16 | 2 |
| 54 | -4 | 0.75 | -10.7 | 2 |
| 55 | -4 | 0.25 | -48 | 3 |
| 56 | -4 | 0.5 | -24 | 3 |
| 57 | -4 | 0.75 | -16 | 3 |
| 58 | -4 | 0.25 | -64 | 4 |
| 59 | -4 | 0.5 | -32 | 4 |
| 60 | -4 | 0.75 | -21.3 | 4 |
| 61 | -5 | 0.25 | -2 | 0.1 |
| 62 | -5 | 0.5 | -1 | 0.1 |
| 63 | -5 | 0.75 | -0.7 | 0.1 |
| 64 | -5 | 0.25 | -6 | 0.3 |
| 65 | -5 | 0.5 | -3 | 0.3 |
| 66 | -5 | 0.75 | -2 | 0.3 |
| 67 | -5 | 0.25 | -10 | 0.5 |
| 68 | -5 | 0.5 | -5 | 0.5 |
| 69 | -5 | 0.75 | -3.3 | 0.5 |
| 70 | -5 | 0.25 | -16 | 0.8 |
| 71 | -5 | 0.5 | -8 | 0.8 |
| 72 | -5 | 0.75 | -5.3 | 0.8 |
| 73 | -5 | 0.25 | -20 | 1 |
| 74 | -5 | 0.5 | -10 | 1 |
| 75 | -5 | 0.75 | -6.7 | 1 |
| 76 | -5 | 0.25 | -26 | 1.3 |
| 77 | -5 | 0.5 | -13 | 1.3 |
| 78 | -5 | 0.75 | -8.7 | 1.3 |
| 79 | -5 | 0.25 | -30 | 1.5 |
| 80 | -5 | 0.5 | -15 | 1.5 |
| 81 | -5 | 0.75 | -10 | 1.5 |
| 82 | -5 | 0.25 | -40 | 2 |
| 83 | -5 | 0.5 | -20 | 2 |
| 84 | -5 | 0.75 | -13.3 | 2 |
| 85 | -5 | 0.25 | -60 | 3 |
| 86 | -5 | 0.5 | -30 | 3 |
| 87 | -5 | 0.75 | -20 | 3 |
| 88 | -5 | 0.25 | -80 | 4 |
| 89 | -5 | 0.5 | -40 | 4 |
| 90 | -5 | 0.75 | -26.7 | 4 |
| 91 | -6 | 0.25 | -2.4 | 0.1 |
| 92 | -6 | 0.5 | -1.2 | 0.1 |
| 93 | -6 | 0.75 | -0.8 | 0.1 |
| 94 | -6 | 0.25 | -7.2 | 0.3 |
| 95 | -6 | 0.5 | -3.6 | 0.3 |
| 96 | -6 | 0.75 | -2.4 | 0.3 |
| 97 | -6 | 0.25 | -12 | 0.5 |
| 98 | -6 | 0.5 | -6 | 0.5 |
| 99 | -6 | 0.75 | -4 | 0.5 |
| 100 | -6 | 0.25 | -19.2 | 0.8 |
| 101 | -6 | 0.5 | -9.6 | 0.8 |
| 102 | -6 | 0.75 | -6.4 | 0.8 |
| 103 | -6 | 0.25 | -24 | 1 |
| 104 | -6 | 0.5 | -12 | 1 |
| 105 | -6 | 0.75 | -8 | 1 |
| 106 | -6 | 0.25 | -31.2 | 1.3 |
| 107 | -6 | 0.5 | -15.6 | 1.3 |
| 108 | -6 | 0.75 | -10.4 | 1.3 |
| 109 | -6 | 0.25 | -36 | 1.5 |
| 110 | -6 | 0.5 | -18 | 1.5 |
| 111 | -6 | 0.75 | -12 | 1.5 |
| 112 | -6 | 0.25 | -48 | 2 |
| 113 | -6 | 0.5 | -24 | 2 |
| 114 | -6 | 0.75 | -16 | 2 |
| 115 | -6 | 0.25 | -72 | 3 |
| 116 | -6 | 0.5 | -36 | 3 |
| 117 | -6 | 0.75 | -24 | 3 |
| 118 | -6 | 0.25 | -96 | 4 |
| 119 | -6 | 0.5 | -48 | 4 |
| 120 | -6 | 0.75 | -32 | 4 |
| 121 | -7 | 0.25 | -2.8 | 0.1 |
| 122 | -7 | 0.5 | -1.4 | 0.1 |
| 123 | -7 | 0.75 | -0.9 | 0.1 |
| 124 | -7 | 0.25 | -8.4 | 0.3 |
| 125 | -7 | 0.5 | -4.2 | 0.3 |
| 126 | -7 | 0.75 | -2.8 | 0.3 |
| 127 | -7 | 0.25 | -14 | 0.5 |
| 128 | -7 | 0.5 | -7 | 0.5 |
| 129 | -7 | 0.75 | -4.7 | 0.5 |
| 130 | -7 | 0.25 | -22.4 | 0.8 |
| 131 | -7 | 0.5 | -11.2 | 0.8 |
| 132 | -7 | 0.75 | -7.5 | 0.8 |
| 133 | -7 | 0.25 | -28 | 1 |
| 134 | -7 | 0.5 | -14 | 1 |
| 135 | -7 | 0.75 | -9.3 | 1 |
| 136 | -7 | 0.25 | -36.4 | 1.3 |
| 137 | -7 | 0.5 | -18.2 | 1.3 |
| 138 | -7 | 0.75 | -12.1 | 1.3 |
| 139 | -7 | 0.25 | -42 | 1.5 |
| 140 | -7 | 0.5 | -21 | 1.5 |
| 141 | -7 | 0.75 | -14 | 1.5 |
| 142 | -7 | 0.25 | -56 | 2 |
| 143 | -7 | 0.5 | -28 | 2 |
| 144 | -7 | 0.75 | -18.7 | 2 |
| 145 | -7 | 0.25 | -84 | 3 |
| 146 | -7 | 0.5 | -42 | 3 |
| 147 | -7 | 0.75 | -28 | 3 |
| 148 | -7 | 0.25 | -112 | 4 |
| 149 | -7 | 0.5 | -56 | 4 |
| 150 | -7 | 0.75 | -37.3 | 4 |
|  |  |  |  |  |
| **Losses Ambiguity Trials** | | | | |
| Trial # | Certain Value  ($) | pWIN  (%) | Gamble Value  ($) | rEV |
| 1 | -3 |  | -0.6 | 0.1 |
| 2 | -3 |  | -1.8 | 0.3 |
| 3 | -3 |  | -3 | 0.5 |
| 4 | -3 |  | -4.8 | 0.8 |
| 5 | -3 |  | -6 | 1 |
| 6 | -3 |  | -7.8 | 1.3 |
| 7 | -3 |  | -9 | 1.5 |
| 8 | -3 |  | -12 | 2 |
| 9 | -3 |  | -18 | 3 |
| 10 | -3 |  | -24 | 4 |
| 11 | -4 |  | -0.8 | 0.1 |
| 12 | -4 |  | -2.4 | 0.3 |
| 13 | -4 |  | -4 | 0.5 |
| 14 | -4 |  | -6.4 | 0.8 |
| 15 | -4 |  | -8 | 1 |
| 16 | -4 |  | -10.4 | 1.3 |
| 17 | -4 |  | -12 | 1.5 |
| 18 | -4 |  | -16 | 2 |
| 19 | -4 |  | -24 | 3 |
| 20 | -4 |  | -32 | 4 |
| 21 | -5 |  | -1 | 0.1 |
| 22 | -5 |  | -3 | 0.3 |
| 23 | -5 |  | -5 | 0.5 |
| 24 | -5 |  | -8 | 0.8 |
| 25 | -5 |  | -10 | 1 |
| 26 | -5 |  | -13 | 1.3 |
| 27 | -5 |  | -15 | 1.5 |
| 28 | -5 |  | -20 | 2 |
| 29 | -5 |  | -30 | 3 |
| 30 | -5 |  | -40 | 4 |
| 31 | -6 |  | -1.2 | 0.1 |
| 32 | -6 |  | -3.6 | 0.3 |
| 33 | -6 |  | -6 | 0.5 |
| 34 | -6 |  | -9.6 | 0.8 |
| 35 | -6 |  | -12 | 1 |
| 36 | -6 |  | -15.6 | 1.3 |
| 37 | -6 |  | -18 | 1.5 |
| 38 | -6 |  | -24 | 2 |
| 39 | -6 |  | -36 | 3 |
| 40 | -6 |  | -48 | 4 |
| 41 | -7 |  | -1.4 | 0.1 |
| 42 | -7 |  | -4.2 | 0.3 |
| 43 | -7 |  | -7 | 0.5 |
| 44 | -7 |  | -11.2 | 0.8 |
| 45 | -7 |  | -14 | 1 |
| 46 | -7 |  | -18.2 | 1.3 |
| 47 | -7 |  | -21 | 1.5 |
| 48 | -7 |  | -28 | 2 |
| 49 | -7 |  | -42 | 3 |
| 50 | -7 |  | -56 | 4 |
